# Effects of Dietary Protein Levels on Bamei Pig Intestinal Colony Compositional Traits

**DOI:** 10.1155/2020/2610431

**Published:** 2020-11-23

**Authors:** Dong Wang, Guoshun Chen, Lili Song, Mingjie Chai, Yongfeng Wang, Shengzhang Shui, Hua Zhang, Yuzhu Sha, Yueyang Yao

**Affiliations:** College of Animal Science and Technology, Gansu Agricultural University, Lanzhou 730070, China

## Abstract

Diets containing different crude protein levels (16%, 14%, and 12%) were created to feed Bamei pigs in order to study the effect of these compositions on intestinal colonies. Therefore, 27 healthy Bamei pigs of similar weight (20.99 kg ± 0.16 kg) were selected and randomly divided into three groups for microbial diversity analysis. The results of this study show that microbial diversities and abundances in Bamei pig jejunum and caecum samples after feeding with different dietary protein levels were significantly different. Dietary crude protein level exerted no significant effect on the Shannon index for cecum microbes in these pigs, while Simpson, ACE, and Chao1 indices for group I were all significantly higher than those of either the control group or group II (*P* < 0.05). Indeed, data show that microbial diversities and abundances in the 14% protein level group were higher than those in either the 16% or 12% groups. Dominant bacteria present in jejunum and cecum samples given low-protein diets were members of the phyla *Firmicutes* and *Bacteroidetes*. Data show that as dietary crude protein level decreases, representatives of the microbial flora genus *Lactobacillus* in jejunum and cecum samples gradually increases. Values for the KEGG functional prediction of microbial flora at different dietary protein levels also show that genes of jejunum and cecum microorganisms were mainly enriched in the “metabolism” pathway and indicate that low protein diets increase intestinal metabolic activity. Therefore, we recommend that Bamei pig dietary protein levels are reduced 2% from their existing level of 16% crude protein. We also suggest that essential synthetic amino acids (AA) are added to optimize this ideal protein model as this will increase intestinal flora diversity in these pigs and enhance health. These changes will have a positive effect in promoting the healthy growth of Bamei pigs.

## 1. Introduction

The animal intestine is the main place where nutrients are digested. A body provides microorganisms with the nutrients they need for growth via the intestine; microorganisms in intestines decompose food materials and produce smaller molecules that the host organism can digest and absorb. Microorganisms in the guts of animals are therefore mutually beneficial [1, 2]; a large number of microorganisms live in animal intestines, reaching a total such that the number of microbial cells exceeds other components [3]. Microorganisms influence host growth, development, nutrient absorption capacity, and intestinal immunity and play a vital role in health [2]. In earlier work, Kim and Isaacson [4] showed that intestinal microorganisms exert a certain promotional effect on animal body growth and development and participate in digestive immune functions. Intestinal microbial diversity therefore exerts an important impact on microbial flora stability. In other words, the higher the diversity, the more stable a flora will be and so the healthier the animal gut. The microbial ecosystem of a pig gastrointestinal tract is influenced by numerous factors but changes in dietary structure are thought to be amongst the most important. The amount and quality of protein in a diet exerts a significant influence on microbial community structure in an intestine [5]; indeed, an intestinal flora seems to be sensitive to source changes in dietary protein. In one example, the use of highly digestible protein sources has been shown to reduce fermentation and the growth of potentially pathogenic species [5]. Similarly, Fan [6] showed that reducing dietary protein levels by just 3% can increase ileal microbial diversity in fattening pigs, and, indeed, when this level is reduced by another 3%, microbial diversity is significantly reduced. In another study, Chen found that restricting 15% of dietary protein can increase the ratio of beneficial microorganisms to harmful bacteria and optimize the structure of the intestinal microbial community [7]. It is clear that dietary protein level plays a very important role as a component of pig production. A correct level not only provides the body with the nutritional effects of amino acids (AA) necessary for endogenous protein synthesis but also participates in regulating feed intake, lipids, and glucose metabolism to maintain growth, development, biological proliferation, digestive enzymes, and hormone secretion in the intestine [8–10].

The demand for protein feed is very large throughout pig production. Indeed, in order to reduce heavy nitrogen emissions into the environment, protein levels should be reduced to promote the sustainable development of this industry [11, 12]. At the same time, the above studies have found that the intestinal flora plays a very important role in the growth and development of pigs, reducing the protein content in the feed, thereby changing the structure of the intestinal flora and enabling the fattening pigs to grow and develop better. Protein levels fed to Bamei pigs were reduced in this study by around 2% and 4% compared with the base protein level of 16%, while essential AA were supplemented. The effects of dietary protein levels on the intestinal microflora of Bamei pigs were studied in order to provide a theoretical basis for further enhancing the production of this species.

## 2. Materials and Methods

### 2.1. Ethics Statement

Experiments involving animals were carried out in accordance with regulations for the Administration of Affairs Concerning Experimental Animals (Ministry of Science and Technology, China; revised in June 2004). Sample collection was carried out according to the guidelines of the Ethics Committee for the Care and Use of Laboratory Animals of Gansu Agricultural University.

### 2.2. Experimental Design and Feeding Management

A total of 27 Bamei pigs with similar weights (21 kg ± 0.15 kg) and good levels of health were selected and randomly divided into three treatments at the Gansu Provincial Farming and Animal Husbandry Breeding Farm, Jingtai County, Gansu Province, China. Three replicates per treatment were carried out with three pigs per replicate (Table 1). The test period used spanned June 24, 2019, to November 20, 2019, encompassing a pretrial period of seven days and a normal trial period of 150 days. The pig house was thoroughly cleaned before the test and disinfected. Throughout the test period, pigs were immunized at the times specified by the farm and were able to freely drink water and eat. The management conditions for each group were the same; disinfection was carried out every Friday, and the pig house was cleaned at 09 : 00 and 16 : 00 each day.

### 2.3. Dietary Composition and Nutrition Level

A basic diet was prepared according to the stage NRC (2012) standard (i.e., between 60 kg and 110 kg) (Table 2), and the nutrient levels in the basic diet are shown in the additional file (Table S1).

### 2.4. Sample Collection and 16S Ribosomal RNA (rRNA) High-Throughput Sequencing

Cecum and jejunum contents were quickly collected in 5 ml cryotubes following slaughter in each case. Intestinal contents samples were collected in five tubes and quickly placed in liquid nitrogen to be stored for subsequent intestinal 16S rRNA microbial detection and analysis. Intestinal microbial DNA extraction, detection, PCR amplification, and product purification from different intestinal samples was carried out before 16S rRNA gene sequencing was performed at Nuohe Biotechnology Co., Ltd.

### 2.5. Data Processing

Test data were preliminarily evaluated and sorted using the software Excel 2010 before variance analysis, and multiple data comparisons were performed through the software SPSS 21.0. The Duncan method was used to test the significance of multiple differences; thus, if *P* < 0.05, then a difference was considered significant, while if *P* > 0.05, then nonsignificance was reported. Test data were expressed in the form mean ± standard deviation (SD).

## 3. Results

### 3.1. 16S rRNA Sequencing Results at Different Dietary Protein Levels

High-throughput 16S rRNA sequencing was performed to assess gut microbes in Bamei pigs fed with different levels of dietary protein. A total of 1,667,502 valid sequences were detected in 18 samples from three groups which had an average length of 413 base pairs (bp). Amongst the eight Bamei pig jejunums with different dietary protein levels, nine samples were sequenced to obtain 847,451 pairs of reads. Once double-end reads had been spliced and filtered, 760,071 optimized sequence numbers (Clean Tags) were generated. These Clean Tags were filtered into chimeras to obtain effective sequence numbers (Effective Tags); data show that each sample generates at least 60,806 Effective Tags, 64,386 on average. Similarly, 820,051 pairs of reads were obtained from nine cecal contents samples. This resulted in 767,631 Clean Tags generated after double-end reads were spliced and subjected to filtration. These Clean Tags were then also filtered into chimeras to obtain effective sequence numbers (Effective Tags). Data show that each sample generates at least 54,142 Effective Tags, 61,382 on average (Additional file: Table S2).

### 3.2. Intestinal Microbial Diversity at Different Dietary Protein Levels

**Figure 1:**
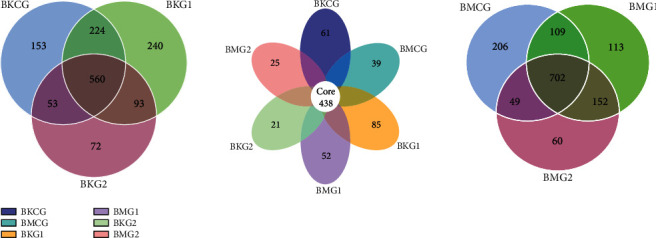
Petal and Venn diagrams for different dietary protein levels.

**Figure 2:**
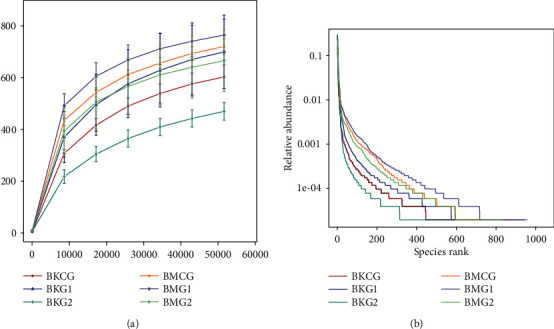
16S rRNA gene sequence dilution curves.

Cluster Operational Taxonomic Units (OTUs) with 97% identity were constructed based on Effective Tags for all samples. It is clear that the OTU petals of the jejunum and cecum span the three protein levels groups (Figure 1). Results reveal 438 OTUs in the jejunum and cecum at different dietary protein levels. In addition, the total number of unique OTUs in the jejunum at three different dietary protein levels was 167, significantly higher than the number in the cecum (116). A Venn diagram of jejunum OTUs at three different protein levels is shown in Figure 1. This presentation shows 560 OTUs present in Bamei pig jejunums at the three different protein levels. The number of OTUs shared by the control group, test group I, and test group II were 224 and 53, respectively, while the number of OTUs shared by test group I and test group II was 93. A Venn diagram of cecal OTUs at three different dietary protein levels can also be seen in Figure 1; this presentation shows that the total number of OTUs is 702, while the total number of OTUs shared between the control group, test group I, and test group II are 109 and 49, respectively. The total number of OTUs in test group I and test group II was 152; thus, unique OTUs present in the control group, test group I, and test group II were 206, 113, and 60, respectively. As shown by the dilution curve also presented in Figure 2, the amount of sequencing data in this study is reasonable as it reflects information about most microorganisms in samples. Results of an Alpha diversity analysis at three different dietary protein levels are presented in Table 3; these data show that microbial diversity and abundances in jejunum and cecum samples from Bamei pigs were different between the three groups. The diversity and abundance of microbes in cecum samples from Bamei pigs were significantly higher than those from jejunum samples (*P* < 0.05). Data show that dietary protein levels exerted no significant effect on Shannon index values for cecum microbes in Bamei pigs, while Simpson, ACE, and Chao1 index values for test I were significantly higher than those recovered for the control group and test group II (*P* < 0.05). Dietary protein levels exerted no significant effect on microbial diversity or abundance in cecum samples from Bamei pigs (*P* > 0.05). Comprehensive test results show that dietary protein levels can improve intestinal microbe numbers in Bamei pigs and also tend to enhance both microbial diversity and abundance (*P* < 0.1).

### 3.3. Classification of Intestinal Microbes at Different Dietary Protein Levels

**Figure 3:**
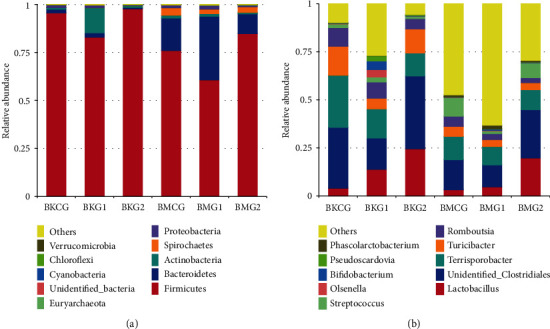
Relative abundance map for microbial groups in jejunum and cecum samples.

The data presented in Figure 3 show that, at the phylum level, bacteria present in jejunum and cecum samples from Bamei pigs are mainly representatives of *Firmicutes* or *Bacteroidetes*. The relative abundance of members of phylum *Firmicutes* at the three protein levels in jejunum samples was 92.1%, while the average relative abundance in cecum samples was 73.88%. Relative abundance values for *Bacteroidetes* and *Actinobacteria* in jejunum samples from test group II were higher than those recovered from the other two groups. Similarly, relative abundances of *Bacteroidetes* in test group II cecum samples were also higher than those of the other two groups; although, this difference is not significant. Selecting microbes at the phylum level for further differential analysis shows that members of four of the top ten were significantly present in jejunum samples. Representatives of phylum *Firmicutes* were present in significantly higher numbers in test group II compared to test group I (*P* = 0.02), while representatives of phylum *Actinobacteria* were present in significantly higher numbers in test group I compared to the control group (*P* = 0.011). Similarly, representatives of phylum *Proteobacteria* were present in significantly higher numbers in the control group than in test group II (*P* = 0.024), while representatives of phylum *Euryarchaeota* were present in significantly higher numbers in test group I than in either the control group or test group II (*P* = 0.001). Data for all other phyla reveal no significant differences, while two from cecum sample did vary. Representatives of phylum *Firmicutes* were present in significantly higher numbers in test group II than in test group I (*P* = 0.034), while representatives of phylum *Bacteroidetes* were present in significantly higher numbers in test group I compared to test group II (*P* = 0.011). Data for other phyla show no significant differences. Comparing microbes at the phyla level in jejunum and cecum samples shows that representatives of phylum *Firmicutes* in former samples occurred at significantly higher levels than in the cecum (*P* = 0.032). Representatives of phylum *Bacteroidetes* in the cecum were also significantly more abundant than in the jejunum (*P* = 0.070), while data for other phyla show no clear differences (*P* > 0.05).

Bacteria in jejunum and cecum samples at the genus level given different dietary protein levels mainly include unidentified *Clostridiales*, *Terrisporobacter*, and *Turicibacter*. Analysis of species differences shows that just the genus *Pseudoscardovia* in the jejunum varied significantly; samples from test group I contain this genus at significantly higher levels than the control group and test group II (*P* = 0.079). Data for other phyla did not vary significantly. Overall, three significant differences in pig cecum contents were seen; *unidentified Clostridiales* composition in test group II samples was significantly higher than test group I (*P* = 0.029), while genus *Romboutsia* contents in the control group was significantly higher than in test group I. Composition of *Streptococcus* in the control group (*P* = 0.030) was also significantly higher than in test group I samples (*P* = 0.079). Comparing microbial differences at the genus level between jejunum and cecum samples, it is clear that representatives of *unidentified_Clostridiales* in the former occurred at significantly higher proportions than in the latter (*P* = 0.021). Differences between other phyla were not significant.

### 3.4. Gene Functional Predictions

**Figure 4:**
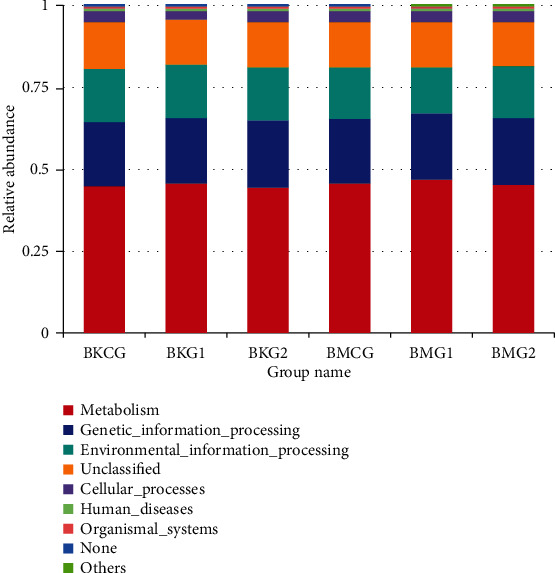
KEGG database functional predictions.

Functional predictions based on the KEGG database at Level 1 suggest that genes in jejunum and cecum microorganisms at different dietary protein levels are mainly enriched in the “metabolism pathway,” followed by “genetic-information-processing” and “environmental-information-processing” pathways. In contrast, at Level 2, the main enrichment pathways are “membrane-transport” and “carbohydrate-metabolism.” No significant differences in microbial gene KEGG pathways were seen in jejunum samples at different dietary protein levels (Figure 4). In contrast, significant differences in KEGG gene enrichment pathways were seen in cecum samples at the three different dietary protein levels (*P* < 0.05); these included “metabolism.” “environmental-information-processing,” and “organismal systems.” Similarly, comparing jejunum and cecum microbial KEGG functional predictions at the three different dietary protein levels reveals that enrichment pathways are significantly different, specifically “metabolism” and “environment-information-processing.”

## 4. Discussion

### 4.1. The Effect of Different Dietary Protein Levels on Intestinal Microflora Diversity

Animal intestines comprise extensive microecological environments, and as organisms rely on their microbial floras to digest and absorb nutrients [13]. The intestine therefore not only provides a suitable living environment for the intestinal flora but also plays a key role in the digestion of food and thus overall health [14]. This means that the number and types of microbes in an intestinal flora play important roles in animal health and growth [2, 5]. In the research on the human gastrointestinal tract, it is also found that the intestinal microbial community is closely related to the energy metabolism, immunity, and oxidative stress of the human body, which in turn affects human health [15]. Diets containing low protein levels can increase the richness and diversity of intestinal floras in finishing pigs. The analysis of differences in bacterial community composition presented here shows that pigs in a diet group with low protein levels had a different gut microflora compared with those in the normal protein level group. Low-protein diets can enhance the intestinal flora balance and benefit overall health, while an excessive protein intake will cause fermentation and produce harmful substances, including ammonia, amines, and phenols. These can adversely influence the intestinal microflora and harm overall health [5, 16, 17], and the harmful substances produced will further harm our living environment. In earlier work, Greenhill [18] and Shoaie et al. [19] both showed that the intestinal microflora of fattening pigs is mainly comprised of representatives within the phyla *Firmicute* and *Bacteroides*. This means that the ratio between these two groups can be used as an indicator to assess some obesity diseases; the higher the ratio, the easier it is for a pig to become obese. At the same time, Shannon and Simpson indices are usually used to assess intestinal microfloral diversity. These values are usually positively correlated with microbial flora stability and ability to resist pathogenic bacterial infections [20]. Results show that both the diversity and abundance of intestinal microbes in Bamei pigs at the 14% dietary protein level increased, while Shannon index values for this group were significantly higher in the jejunum and cecum than for the basal protein level group (16%). These results show that changes in dietary protein level are beneficial to the intestinal health of Bamei pigs and can increase both the richness and diversity of intestinal flora in growing and finishing animals. Previous results published by Rist et al. [21] showed that feeding at different protein levels can influence small intestine microbial composition and promote the reproduction of beneficial bacteria, thereby improving intestinal health. Shows that dietary changes can lead to changes in the gut microbial environment, thereby affecting the health of the host. Similar findings have been found in human studies [22–25]. Cao et al. [26] studied the effects of dietary protein on the intestinal microbes of weaned piglets; the results of this study showed that increasing dietary protein level also enhanced the number and types of intestinal microbes in weaned piglets as well as their intestinal microenvironments [27]. Fan et al. [20] also showed that when dietary protein level decreased from 16% to 13%, the abundance and diversity of ileal flora increased alongside the proportion of the beneficial *Lactobacillus*. Son et al. also found that changes in dietary structure led to the increase of beneficial *Lactobacillus* in the athletes' intestines [25], which indicates that dietary changes have made the intestinal flora more abundant and beneficial flora increased, which is more conducive to the growth and development of pigs. The proportion of colonic beneficial bacteria *Megacoccus* also increased significantly. In contrast, when dietary protein level fell to 10%, the diversity of ileum and colon flora in growing and finishing pigs decreased in concert with their growth performance. These results show that changes in dietary protein levels can promote microbial diversity in the intestine of Bamei pigs to a certain extent, but an excessive reduction will influence normal growth performance. Thus, under normal circumstances, a gut microflora changes within a certain physiological range given fluctuations in the external environment and diet [28]. Studies have shown that when dietary protein is reduced from 23% by 4% or more, piglet the performance can compromised. Low-protein diets also maintain pig intestinal health by reducing toxic microbial metabolites and improve microbial diversity [29]. The microbial diversity analysis presented here shows that the communities seen in different protein groups have obvious distributions in the jejunum and cecum. Microorganisms at the 14% level grouped well which indicates that diets containing low protein levels can significantly improve the intestinal microflora of Bamei pigs.

### 4.2. Effects of Different Dietary Protein Levels on Intestinal Microflora Structure and KEGG Functional Predictions

Analyzing the structural composition of animal intestinal microbes can reflect growth environments and diet to a certain extent [30]. In one study, Zhou et al. [31] found that low-protein diets can regulate microbial composition and metabolites in pig hindguts without influencing growth performance; although, the potential effects of this regulation on health remains unknown. Here, microbes in the jejunum and cecum of Bamei pig were mainly shown to comprise representatives of phyla *Firmicutes* and *Bacteroidetes*; although, significant differences were seen between groups at different protein levels. Representatives of phylum *Firmicutes* in jejunum test group II samples occurred at significantly higher proportions than those in test group I samples (*P* = 0.020). Similarly, representatives of phylum *Firmicutes* in caecum test group II samples also occurred at significantly higher levels than in test group I (*P* = 0.034), while representatives of phylum *Bacteroidetes* in test group I samples also occurred at significantly higher levels than in test group II samples (*P* = 0.011). These outcomes show that reducing the protein level can have an effect on the structure of the main flora of the phylum level. Indeed, *Firmicutes* is also the dominant intestinal phylum present in the low protein group, consistent with previous studies [7, 13]. Dai et al. [32] showed that, at the genus level, bacteria in the jejunum of Bamei pigs mainly comprise unidentified *Clostridiales*, *Terrisporobacter*, and *Turicibacter*, while those in the cecum mainly include unidentified *Clostridiales*, *Terrisporobacter*, *Turicibacter*, and *Streptococcus*. The main role of *Streptococcus* appears to be to utilize AA in the intestine to synthesize bacterial proteins. In another earlier study, Fan et al. [20] showed that as dietary protein levels decrease, the abundance of *Clostridium sensu stricto 1* in ileum samples from pigs decreased significantly. The reason for the analysis is that a decrease in dietary protein level causes a reduction in nitrogen source as a fermentation substrate. The relative abundances of *Terrisporobacter* in the diet group with a normal protein level were also significantly higher than in the group fed a low protein level; this genus is an emerging anaerobic pathogen, but the few cases published so far usually report it as a component of a multimicrobial infection [33]. Similarly, *Romboutsia* occurred at a higher level in the normal diet protein group than the low protein group. The results of this study show that as dietary protein levels decrease, the proportion of Lactobacillus in jejunum and cecum of microbial floras also gradually increased. Dietary protein levels therefore exert a certain influence on the composition of Bamei pig intestinal floras. As dietary protein levels decrease, no significant differences in abundance either the jejunum or caecum were seen; although, this change can improve microbial composition. The outcomes of this study have also enabled us to predict the KEGG functions of genes present in microbial floras at different dietary protein levels. These results show that genes extracted from jejunum and cecum microbes at different dietary protein levels were mainly enriched in the “metabolism pathway,” followed by “genetic-information-processing,” and “environmental-information-processing” channels. In contrast, at Level 2, the main enrichment pathways are “membrane-transport” and “carbohydrate-metabolism.” Enrichment pathways in jejunum and cecum genes are significantly different in terms of the presence of “metabolism” and “environmental-information-processing”; these results indicate that an appropriate reduction in dietary protein can have a beneficial effect on the metabolic function of microorganisms in the intestine. A reduction can assist metabolic activities, consistent with previous work [34, 35].

## 5. Conclusions

The experimental results presented here show that when a 16% crude protein Bamei pig basic diet was reduced by between 2% and 4% with corresponding EAA additions, significant differences in jejunum and cecum microbes were detected. A reduction in dietary protein can enhance microbial composition and flora in both the cecum and jejunum, promote the growth of beneficial bacteria, and have an overall positive effect on Bamei pig health.

## Figures and Tables

**Table 1:** Dietary protein level.

Project	Control group	Test I	Test II
Dietary protein level (%)	16.0	14.0	12.0

**Table 2:** Basic diet compositions and nutritional levels.

Basic diet composition (%)				
Raw material	Control group	Test I	Test II	Raw material price (RMB/kg)
Corn	63.50	68.00	72.00	1.96
Soybean meal	18.30	12.90	7.20	3.36
Wheat bran	5.00	5.00	5.00	1.64
Alfalfa meal	5.00	6.50	9.00	1.40
Bentonite	4.00	2.90	1.60	0.43
Soybean oil	1.50	1.70	2.00	6.35
Compound enzyme preparation	0.10	0.10	0.10	15.00
Lys (98%)	0.09	0.23	0.37	8.60
Met (98%)	—	0.03	0.05	20.55
Thr (98%)	—	0.08	0.15	11.00
Trp (98%)	—	0.03	0.05	86.00
CaCO3	0.51	0.46	0.30	0.19
Ca(HCO3)2	1.15	1.22	1.33	2.08
0.5% fattening pig core feed ①	0.50	0.50	0.50	5.80
Feed grade sodium chloride	0.35	0.35	0.35	0.56
Sum	100.00	100.00	100.00	—
Recipe cost (Yuan/kg)	2,202.519	2,192.415	2,170.789	—
Comparison with control group formulation cost	0	–10.10	–31.73	—

**Table 3:** The effects of dietary protein levels on intestinal microbiota diversities in Bamei pigs.

Project	Diversity index		Richness index		Coverage
	Shannon index	Simpson index	ACE index	Chao1 index	
BKCG	3.53 ± 0.15^c^	0.79 ± 0.04^b^	698.14 ± 77.83^ab^	713.96 ± 89.93^ab^	0.997
BKG1	4.59 ± 0.78*b*^c^	0.89 ± 0.04^a^	803.32 ± 165.83^a^	810.78 ± 161.73^a^	0.997
BKG2	3.42 ± 0.47^c^	0.82 ± 0.06^b^	575.07 ± 43.60^b^	590.78 ± 32.89^b^	0.997
BMCG	5.99 ± 0.16^a^	0.95 ± 0.01^a^	813.46 ± 66.24^a^	840.38 ± 88.98^a^	0.997
BMG1	6.34 ± 0.67^a^	0.96 ± 0.03^a^	839.73 ± 70.83^a^	840.73 ± 69.58^a^	0.998
BMG2	5.18 ± 1.04^ab^	0.91 ± 0.04^a^	748.88 ± 139.85^ab^	751.03 ± 143.13^ab^	0.998
P value	0.121	0.094	0.150	0.204	

Note: values in the same column with different small letter superscripts are significantly different (*P* < 0.05), while those with the same, or no, letter superscripts are significantly different (*P* > 0.05).

## Data Availability

All relevant data in this study are presented in the Results section.

## Supplementary Materials

Supplementary Materials Additional file. Table S1: Nutrient levels in basic diets. Table S2. Jejunum sample sequence statistics.
