# Supplementary material for: Effects of Dietary Protein Levels on Bamei Pig Intestinal Colony Compositional Traits
Source: Biomed Res Int. 2020 Nov 23;2020:2610431. doi: 10.1155/2020/2610431 (PMC7714570; doi:10.1155/2020/2610431)
Supplement: Supplementary Materials — Additional file. Table S1: Nutrient levels in basic diets. Table S2. Jejunum sample sequence statistics. [file 2610431.f1.pdf]

Table S1 Nutrient levels in basic diets②

Project	Control group	Test I	Test II
DE (MJ/kg)	13.14	13.13	13.11
CP (%)	15.94 (16.00%) ③	14.00 (14.00%) ③	12.05 (12.00%) ③
CF (%)	3.22	3.50	4.02
Ca (%)	0.60	0.61	0.60
P (%)	0.55	0.55	0.55
Na+（%）	0.16	0.16	0.17
Cl-（%）	0.27	0.27	0.28
SIDLys (%)	0.86	0.86	0.86
SIDMet (%)	0.26	0.26	0.26
SIDThr (%)	0.59	0.59	0.59
SIDTrp (%)	0.19	0.19	0.19

Note: ① Added to the diet per kg of premix: Fe, 64.00 mg; Zn, 71.00 mg; Mn, 35.00 mg; Cu, 17.00 mg; Se, 0.36 mg; I, 0.64 mg; Vitamin A, 790 IU; Vitamin D3 135 IU; Vitamin E, 55.00 mg; Thiamine (Vitamin B1), 2.20 mg; Riboflavin (Vitamin B2), 2.50 mg; Biotin, 0.05 mg; Folic acid, 0.35 mg; Nicotinic acid, 29.00 mg; Calcium pantothenate, 27.00 mg Vitamin B6, 0.09 mg; Vitamin B 12, 1.00 mg; Choline, 5,000 mg; Flavoring agent, 3,000 mg; Sweetener, 3,000 mg; Phytase, 4,000 mg; Lysine, 30,000 mg; Tryptophan, 2,000 mg;

② Nutritional components are expressed as calculated values;

③ Test set value in brackets.

Table S2 Jejunum sample sequence statistics

Group	Sample serial number	PE Reads		Clean Tags		Effective Tags		Effective (%)	
		Jejunum	Cecum	jejunum	Cecum	Jejunum	Cecum	Jejunum	Cecum
Control group BKCG/BMCG	BKCG1/BMCG1	97,644	95,857	81,189	88,156	62,543	62,924	64.05	65.64
	BKCG2/BMCG2	92,814	81,853	82,566	76,576	63,243	54,142	68.14	66.15
	BKCG3/BMCG3	85,016	95,978	79,167	90,756	62,441	63,407	73.45	66.06
Test Ⅰ BKG1/BMCG1	BKG11/BMCG11	97,213	91,096	81,053	83,811	67,312	60,700	69.24	66.63
	BKG12/BMCG12	95,987	95,647	84,550	89,824	67,073	63,977	69.88	66.89
	BKG13/BMCG13	99,623	82,905	91,840	77,267	66,283	61,474	66.53	74.15
Test Ⅱ BKG2/BMCG2	BKG21/BMCG21	83,398	99,293	78,064	93,368	62,903	64,121	75.43	64.58
	BKG22/BMCG22	98,771	82,758	91,246	77,831	60,806	60,644	61.56	73.28
	BKG23/BMCG23	96,985	94,664	90,396	90,042	66,871	61,047	68.95	64.49
